# Prognostic significance of cancer family history for patients with gastric cancer: a single center experience from China

**DOI:** 10.18632/oncotarget.9032

**Published:** 2016-04-26

**Authors:** Xiaowen Liu, Hong Cai, Lin Yu, Hua Huang, Ziwen Long, Yanong Wang

**Affiliations:** ^1^ Department of Gastric Cancer and Soft Tissue Sarcoma Surgery, Fudan University Shanghai Cancer Center, Shanghai, China; ^2^ Department of Oncology, Shanghai Medical College, Fudan University, Shanghai, China; ^3^ Department of Pathology, Fudan University Shanghai Cancer Center, Shanghai, China

**Keywords:** gastric cancer, prognosis, cancer family history

## Abstract

Family history of cancer is a risk factor for gastric cancer. In this study, we investigated the prognoses of gastric cancer patients with family history of cancer. A total of 1805 gastric cancer patients who underwent curative gastrectomy from 2000 to 2008 were evaluated. The clinicopathologic parameters and prognoses of gastric cancer patients with a positive family history (PFH) of cancer were compared with those with a negative family history (NFH). Of 1805 patients, 382 (21.2%) patients had a positive family history of cancer. Positive family history of cancer correlated with younger age, more frequent alcohol and tobacco use, worse differentiation, smaller tumor size, and more frequent tumor location in the lower 1/3 of the stomach. The prognoses of patients with a positive family history of cancer were better than that of patients with a negative family history. Family history of cancer independently correlated with better prognosis after curative gastrectomy in gastric cancer patients.

## INTRODUCTION

Despite decreasing incidence and mortality, gastric cancer remains the fifth most common cancer and the third most common cause of cancer-related death worldwide [1]. A number of environmental factors are correlated with gastric cancer development [2–5]. Additionally, a family history of cancer, especially gastric cancer, is associated with increased risk of developing the disease [6, 7]. It is estimated that approximately 13.5% to 46.4% of gastric cancer patients have a family history of cancer [8–10]. Recently, longer overall survival has been reported in other cancer patients with a family history of cancer [11, 12]. Although some studies have reported the clinicopathological features and prognosis of gastric cancer patients with a family history of cancer, these results have been inconsistent [8, 9, 13, 14]. Therefore, the effect of family history of cancer on survival in gastric cancer patients is still unclear. To clarify this question, we conducted this study to evaluate the correlation between family history of cancer and clinicopathologic characteristics and overall survival of gastric cancer patients.

## RESULTS

### Clinicopathological characteristics

Patients included 1263 males and 542 females (2.3:1) with a mean age of 58 years. There were 339 (18.8%) early gastric cancers and 1466 (81.2%) advanced gastric cancers. Differentiated tumors were observed in 471 (26.1%) patients, and undifferentiated in 1334 (73.9%) patients. 339 (18.8%) were type 0, 9 (0.5%) type I, 502 (27.8%) type II, 879 (48.7%) type III, 76 (4.2%) type IV. Of 1805 patients, 577 (32.0%) had tumors located in the upper third, 298 (16.5%) had tumors in the middle third, 821 (45.5%) had tumors in the lower third of the stomach, and 109 (6.0%) had tumors occupying two-thirds or more of stomach. Lymph node metastasis was observed in 1122 patients (62.2%). The distribution of pathological stage was as follows: 279 (15.5%) patients had stage IA tumors, 216 (12.0%) IB, 186 (10.3%) IIA, 244 (13.5%) IIB, 230 (12.7%) IIIA, 291 (16.1%) IIIB, and 359 (19.9%) IIIC. Patients demographics are listed in Table 1.

**Table 1:** Patient cohort

	*n* =1805	100%
Sex		
Male	1263	70.0
Female	542	30.0
Age (yr)		
<60	997	55.2
≥60	808	44.8
Tumor size (cm)		
<5	902	61.6
≥5	562	38.4
Histological type		
Differentiated	471	26.1
Undifferentiated	1334	73.9
Tumor location		
Upper third	577	32.0
Middle third	298	16.5
Lower third	821	45.5
Two-third or more	109	6.0
Borrmann type		
0	339	18.8
I	9	0.5
II	502	27.8
III	879	48.7
IV	76	4.2
Vascular tumor emboli		
Yes	620	34.3
No	1185	65.7
Nervous invasion		
Yes	657	36.4
No	1148	63.6
Pathological stage		
IA	279	15.5
IB	216	12.0
IIA	186	10.3
IIB	244	13.5
IIIA	230	12.7
IIIB	291	16.1
IIIC	359	19.9
Family history of cancer		
Positive	382	21.2
Negative	1423	78.8
Smoking		
Yes	193	10.7
No	1612	89.3
Drinking		
Yes	127	7.0
No	1678	93.0
P21 expression		
Positive	1134	62.8
Negative	671	37.2
P53 expression		
Positive	1319	73.1
Negative	486	26.9
c-myc expression		
Positive	1138	63.0
Negative	667	37.0
EGFR expression		
Positive	697	38.6
Negative	1108	61.4
Neu/Her-2		
Positive	43	2.4
Negative	1762	97.6

### Immunohistochemical characteristics

The expression of p21, p53, c-myc, EGFR and Neu/Her-2 was examined by immunohistochemical staining. The location of staining was predominantly in the cell nucleus for p21 and p53, cell cytoplasm for c-myc, cell cytoplasm or membrane for EGFR, and membrane for Neu/Her-2. The positive expression rates of p21, p53, c-myc, EGFR, and Neu/Her-2 were 62.8%, 73.1%, 63.0%, 38.6%, and 2.4%, respectively.

### Family history of cancer

Of 1805 patients, 382 (21.2%) had at least one relative with any type of cancer. By cancer type, gastric cancer was the most common and occurred in 190 patients (10.5%), while 192 patients (10.6%) had a family history of other cancers. 348 (19.3%) patients had a family history in first-degree relatives, and 34 (1.9%) in second-degree relatives. In the patients with a family history of gastric cancer, 169 (9.4%) had a family history in first-degree relatives and 21 (1.2%) in second-degree relatives. Data is shown in Table 2.

**Table 2:** Family histories of cancer in gastric cancer patients

Family history	No. of patients (1805)	%
Cancer		
Yes	382	21.2
No	1423	78.8
Relatives		
First degree	348	19.3
Second degree	34	1.9
No. of relatives with cancer		
1	258	14.3
≥2	124	6.9
Cancer type		
Gastric cancer	190	10.5
All other cancers	192	10.6
Gastric cancer		
Yes	190	10.5
No	1615	89.5
Relatives		
First degree	169	9.4
Second degree	21	1.2
No. of relatives with gastric cancer		
1	113	6.3
≥2	77	4.3
All other cancers without gastric cancer		
Yes	192	10.6
No	1613	89.4
Relatives		
First degree	179	9.9
Second degree	13	0.7
No. of relatives with cancer		
1	145	8.0
≥2	47	2.6

### Demographic and clinicopathologic features of PFH

Demographically, patients with a positive family history of cancer were younger than patients without positive family history of cancer. There was no difference in gender distribution between the two groups. In patients with a positive family history of cancer, the proportion of smoking and alcohol use was higher than in patients without family history. Clinicopathologically, significant differences were observed in degree of differentiation, tumor location, tumor size, and p21 expression between the two groups. Patients with a positive family history of cancer had a higher rate of undifferentiated tumors and lower 1/3 tumors, smaller tumors, and a lower rate of p21 expression than in those without a positive family history. Data were shown in Table 3.

**Table 3:** Comparison of the clinicopathological characteristics of patients with positive family history of cancer (PFH) and negative family history of cancer (NFH)

Variables	PFH *n*= 382	NFH *n*= 1423	*P*
Gender			0.297
Male	259	1004	
Female	123	413	
Age (yr)			0.0003
<60	242	755	
≥60	140	668	
Tumor size (cm)			0.007
<5	267	889	
≥5	115	534	
Histological type			0.002
Differentiated	76	395	
Undifferentiated	306	1028	
Tumor location			0.021
Upper third	97	480	
Middle third	67	231	
Lower third	192	629	
Two-third or more	26	83	
Borrmann type			0.088
0	88	251	
I	3	6	
II	93	409	
III	180	699	
IV	18	58	
Vascular tumor emboli			0.483
Yes	137	483	
No	245	940	
Nervous invasion			0.152
Yes	151	506	
No	231	917	
Pathological stage			0.207
IA	73	206	
IB	47	169	
IIA	37	149	
IIB	51	193	
IIIA	47	183	
IIIB	48	243	
IIIC	79	280	
Smoking			<0.001
Yes	70	123	
No	312	1300	
Drinking			0.001
Yes	41	86	
No	341	1337	
P21 expression			0.012
Positive	219	915	
Negative	163	508	
P53 expression			0.985
Positive	279	1040	
Negative	103	383	
c-myc expression			0.158
Positive	229	909	
Negative	153	514	
EGFR expression			0.066
Positive	132	565	
Negative	250	858	
Neu/Her-2			0.054
Positive	4	39	
Negative	378	1384	

### Univariate analysis

**Figure 1:**
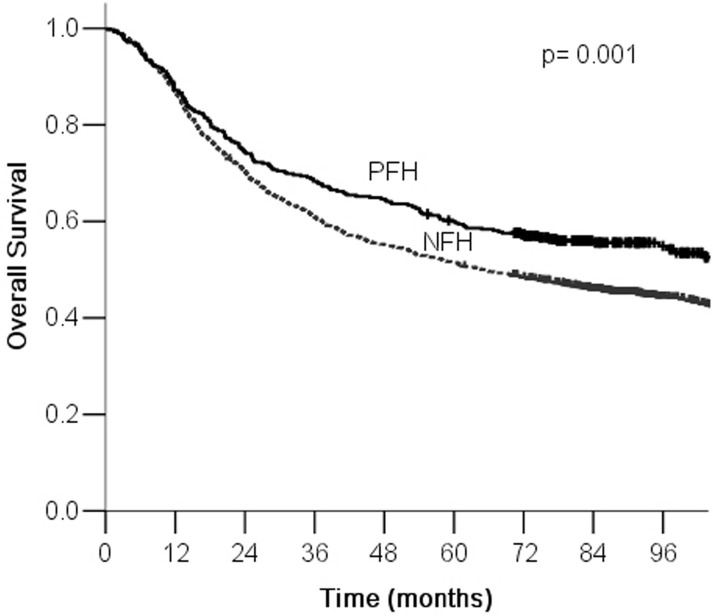
Kaplan-Meier survival curves by family history of cancer There were significant differences between PFH and NFH.

The overall 5-year survival rate was 53% for all patients. The 5-year survival rates of PFH and NFH groups were 60% and 52%, and the difference was statistically significant (Figure 1). Additionally, significant prognostic factors included age, differentiation, vascular tumor emboli, nervous invasion, tumor location, tumor size, Borrmann type, TNM stage, family history of gastric cancer, family history of other cancers, p21 overexpression, Neu/Her-2 overexpression, and EGFR overexpression (Table 4). In the PFH group, vascular tumor emboli, nervous invasion, tumor location, tumor size, Borrmann type, TNM stage, p21 overexpression, and c-myc overexpression were significant prognostic factors for survival (Table 5). In the NFH group, age, differentiation, venous tumor emboli, nervous invasion, tumor location, tumor size, Borrmann type, TNM stage, p21 overexpression, Neu/Her-2 overexpression, and EGFR overexpression were significantly correlated with prognosis (Table 6).

**Table 4:** Univariate analysis of all patients by Kaplan-meier method

Variable	*n*	5-Year survival rate (%)	*P* value
Sex			0.659
Male	1263	52.4	
Female	542	55.4	
Age (yr)			<0.001
<60	997	58.9	
≥60	808	46.4	
Tumor size (cm)			<0.001
<5	1156	62.3	
≥5	649	37.4	
Histological type			<0.001
Differentiated	471	62.0	
Undifferentiated	1334	50.3	
Tumor location			<0.001
Upper third	577	41.2	
Middle third	298	51.2	
Lower third	821	66.1	
Two-third or more	109	26.2	
Borrmann type			<0.001
0	339	91.3	
I	9	40.3	
II	502	51.1	
III	879	42.8	
IV	76	21.1	
Vascular tumor emboli			<0.001
Yes	620	31.4	
No	1185	64.8	
Nervous invasion			<0.001
Yes	657	33.4	
No	1148	64.7	
Pathological stage			<0.001
IA	279	93.7	
IB	216	88.6	
IIA	186	66.8	
IIB	244	56.8	
IIIA	230	47.2	
IIIB	291	30.4	
IIIC	359	13.0	
Smoking			0.061
Yes	193	52.5	
No	1612	60.3	
Drinking			0.240
Yes	127	57.9	
No	1678	53.0	
Family history of cancer			0.001
Positive	382	59.8	
Negative	1423	51.6	
Family history of gastric cancer			0.031
Positive	190	54.2	
Negative	1615	43.0	
Family history of other cancers			0.038
Positive	192	54.2	
Negative	1613	43.0	
P21 expression			0.002
Positive	1134	50.5	
Negative	671	58.0	
P53 expression			0.606
Positive	1319	54.0	
Negative	486	51.5	
c-myc expression			0.333
Positive	1138	52.4	
Negative	667	54.8	
EGFR expression			0.006
Positive	697	48.3	
Negative	1108	56.3	
Neu/Her-2			0.019
Positive	43	30.2	
Negative	1762	53.8	

**Table 5:** Kaplan-Meier univariate analysis of patients with PFH

Variable	*n*	5-Year survival rate (%)	*P* value
Sex			0.540
Male	259	57.1	
Female	123	65.8	
Age (yr)			0.380
<60	242	60.7	
≥60	140	58.9	
Tumor size (cm)			<0.001
<5	267	67.9	
≥5	115	41.3	
Histological type			0.160
Differentiated	76	65.9	
Undifferentiated	306	57.9	
Tumor location			<0.001
Upper third	97	50.3	
Middle third	67	61.9	
Lower third	192	68.5	
Two-third or more	26	23.1	
Borrmann type			<0.001
0	88	91.4	
I	3	0.0	
II	93	63.9	
III	180	44.7	
IV	18	33.3	
Vascular tumor emboli			<0.001
Yes	137	37.6	
No	245	71.5	
Nervous invasion			<0.001
Yes	151	41.9	
No	231	71.2	
Pathological stage			<0.001
IA	73	93.8	
IB	47	88.8	
IIA	37	73.3	
IIB	51	60.8	
IIIA	47	58.7	
IIIB	48	41.7	
IIIC	79	13.8	
Smoking			0.833
Yes	70	57.3	
No	312	60.1	
Drinking			0.819
Yes	41	58.5	
No	341	59.9	
P21 expression			0.041
Positive	219	54.9	
Negative	163	66.8	
P53 expression			0.535
Positive	279	61.1	
Negative	103	55.8	
c-myc expression			0.017
Positive	229	54.8	
Negative	153	66.8	
EGFR expression			0.196
Positive	132	53.0	
Negative	250	63.3	
Neu/Her-2			0.545
Positive	4	50.0	
Negative	378	60.1	

**Table 6:** Kaplan-Meier univariate analysis of patients with NFH

Variable	*n*	5-Year survival rate (%)	*P* value
Sex			0.939
Male	1004	51.2	
Female	419	52.4	
Age (yr)			<0.001
<60	755	58.3	
≥60	668	43.8	
Tumor size (cm)			<0.001
<5	889	60.5	
≥5	534	36.5	
Histological type			<0.001
Differentiated	395	60.9	
Undifferentiated	1028	47.8	
Tumor location			<0.001
Upper third	480	39.2	
Middle third	231	47.8	
Lower third	629	65.3	
Two-third or more	83	26.7	
Borrmann type			<0.001
0	251	90.9	
I	6	50.0	
II	409	48.0	
III	699	42.1	
IV	58	17.2	
Vascular tumor emboli			<0.001
Yes	483	29.5	
No	940	62.7	
Nervous invasion			<0.001
Yes	506	30.6	
No	917	62.9	
Pathological stage			<0.001
IA	206	93.4	
IB	169	88.6	
IIA	149	65.2	
IIB	193	55.7	
IIIA	183	43.3	
IIIB	243	28.2	
IIIC	280	12.8	
Smoking			0.050
Yes	123	60.9	
No	1300	50.6	
Drinking			0.334
Yes	86	57.1	
No	1337	51.1	
P21 expression			0.031
Positive	915	49.4	
Negative	508	55.4	
P53 expression			0.781
Positive	1040	52.0	
Negative	383	50.2	
c-myc expression			0.781
Positive	909	51.8	
Negative	514	51.1	
EGFR expression			0.023
Positive	565	47.4	
Negative	858	54.3	
Neu/Her-2			0.037
Positive	39	28.2	
Negative	1384	52.2	

### Multivariate analysis

Multivariate analysis showed that family history of cancer, age, tumor differentiation, vascular tumor emboli, Borrmann type, tumor size, TNM stage, and p21 overexpression were independent prognostic factors for all patients (Table 7). In the PFH group, TNM stage and c-myc overexpression were significant prognostic factors (Table 8). In the NFH group, age, differentiation, vascular tumor emboli, and TNM stage were independent prognostic factors (Table 9).

**Table 7:** Multivariate analysis of patients by Cox model

Variable	*P* value	RR	95% CI
Age	<0.001	1.327	1.170-1.505
Histological type	0.007	1.234	1.060-1.437
Vascular tumor emboli	0.005	1.225	1.065-1.409
Nervous invasion	0.149	1.108	0.964-1.273
Tumor location	0.081	0.944	0.885-1.007
Borrmann type	0.041	1.093	1.004-1.191
Tumor size	0.035	1.149	1.010-1.308
Pathological stage	<0.001	1.464	1.400-1.532
Family history of cancer*	0.033	0.836	0.708-0.986
Family history of gastric cancer*	0.309	0.891	0.714-1.113
Family history of other cancers*	0.073	0.817	0.655-1.019
P21	0.045	1.146	1.003-1.309
EGFR	0.183	1.091	0.960-1.240
Neu/Her-2	0.173	1.287	0.895-1.851

* Only one parameter can be put into Cox proportional hazards model very time.

**Table 8:** Multivariate analysis of patients with PFH

Variable	*P* value	RR	95% CI
Vascular tumor emboli	0.109	1.312	0.942-1.830
Nervous invasion	0.506	1.120	0.802-1.562
Tumor location	0.404	0.934	0.796-1.096
Tumor size	0.165	1.253	0.911-1.724
Borrmann type	0.097	1.184	0.970-1.445
Pathological stage	<0.001	1.452	1.305-1.617
P21	0.094	1.307	0.955-1.787
c-myc	0.028	1.424	1.039-1.953

**Table 9:** Multivariate analysis of patients with NFH

Variable	*P* value	RR	95% CI
Age	<0.001	1.393	1.212-1.601
Histological type	0.005	1.270	1.077-1.499
Vascular tumor emboli	0.019	1.203	1.030-1.405
Nervous invasion	0.182	1.110	0.952-1.293
Tumor location	0.115	0.944	0.880-1.014
Tumor size	0.085	1.133	0.983-1.305
Borrmann type	0.140	1.074	0.977-1.180
Pathological stage	<0.001	1.469	1.397-1.544
P21	0.171	1.108	0.957-1.284
EGFR	0.140	1.112	0.966-1.280
Neu/Her-2	0.188	1.287	0.884-1.876

### Comparison of survival according to stage between PFH and NFH groups

**Figure 2:**
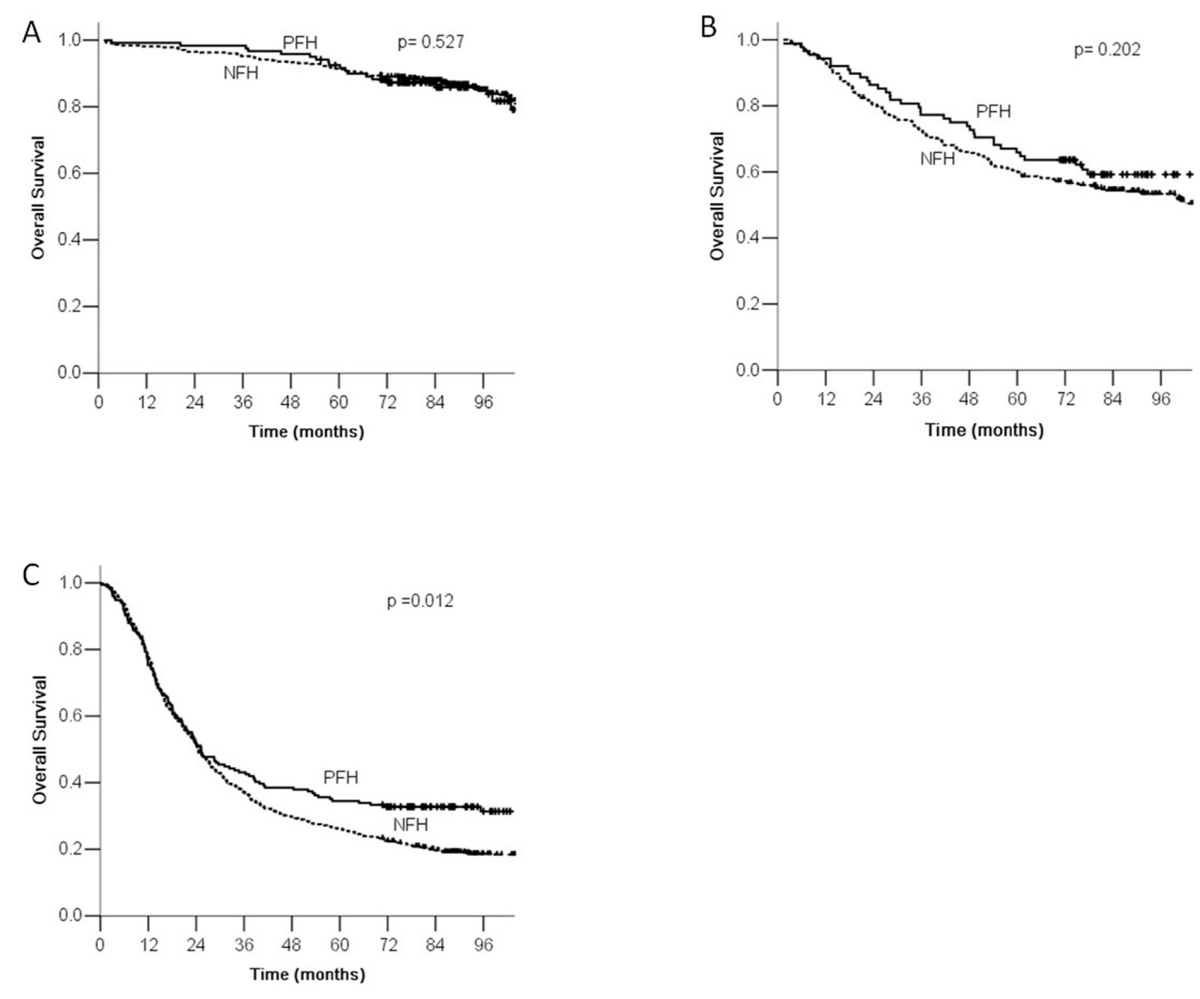
Comparison of survival according to tumor stage There were significant differences between PFH and NFH according to stage III.

According to the AJCC/TNM staging, gastric cancer patients were divided into stage I, stage II, and stage III. According to family history of cancer, each stage was divided into PFH and NFH groups. There was a statistically significant difference in overall survival between the PFH and NFH groups for patients with stage III tumors (*P* <0.05, Figure 2).

## DISCUSSION

Familial aggregation is quite common in all kinds of cancers. In this study, 21.2% of gastric patients had a positive family history of cancer. This is similar to that reported in previous studies [8–10]. The reason for familial aggregation is unclear. It is possible that environmental factors or genetic factors contribute to this. Some studies have shown that environmental factors such as diet or socioeconomic status were significantly associated with risk of family gastric cancer [15, 16]. Additionally, Some studies have reported that microsatellite instability (MSI) was associated with family history of gastric cancer [17, 18]. Lee et al. reported that p53 overexpression may increase familial aggregation of gastric cancer [8]. In the current study, we examined expression of some genes, and we found that p21 expression by tumor cells correlated with family history of gastric cancer. Further study is needed to elucidate the mechanism.

In this study, we found that gastric cancer patients with family history of cancer had different clinicopathological features compared to those without a family history of cancer. Our results showed that patients with a family history of cancer were younger than patients without family history of cancer. However, this result was inconsistent with that reported by a Korean study [9], which found that there was no significant difference in mean ages between familiar gastric cancer and sporadic cancer. It is possible that difference is due to the bias of self-reported family history. Another two recent studies have confirmed our results [19, 20]. Additionally, we found that patients with positive family history of cancer had a higher rate of lower 1/3 tumors. Inoue et al [21] also reported that tumors were more frequently located in the lower and middle part of the stomach in gastric cancer patients with a positive family history. In all, the differences of clinicopathological features and some genes expression between two groups indicated that gastric cancer with positive family history may represent a distinct disease.

Although some studies have reported the effects of a positive family history on the survival of patients with gastric cancer, the results were controversial [8, 9, 13, 14]. These inconsistencies might be due to the adjustment range of confounding variables. Additionally, it might be explained by low statistical power as a result of small-scale sample. In our study, family history of cancer was consistently associated with prognosis in both univariate and multivariate analyses after adjustment for prognostic variables. It is not clear why a family history of cancer increase survival. It is possible that a family history of cancer may heighten awareness of gastric cancer in family members, leading to earlier diagnosis and better prognosis. We found that patients with a positive family history were more likely to have smaller tumor size. However, the current study could not confirm this hypothesis as a result of no information about previous screening. Some studies have shown that patients with a family history of cancer are more likely to undergo cervical cancer and prostate cancer screening [22, 23]. Additionally, health behaviour may also have contributed to the better survival of patients with family history of cancer. Patients with a family history of cancer more likely to have good behavioural habits, like quitting smoking, or healthy dietary habits [24, 25]. Given the fact that smoking and drinking habits are associated with poor prognosis in gastric cancer, a reduced incidence of unhealthy behaviour may partly account for improved prognosis. Han et al. reported that proportions of current smokers or drinkers were significantly lower in patients with a family history of cancer[9]. In contrast, we found that proportions of smokers or drinkers were significantly higher in patients with family history, and smoking or drinking did not affect the survival of gastric cancer patients. Therefore, the effect of health behaviour on prognosis needs further investigation. Finally, genetics may also account for the survival differences of gastric cancer patients with a family history. Microsatellite instability (MSI) is detected frequently in gastric cancer. It has been reported that MSI is associated with a family history of gastric cancer and better overall prognosis [17, 18, 26]. In this study, expressions of p21, p53, c-myc, EGFR and Neu/Her-2 were examined by immunohistochemical staining. We found that rate of p21 expression was lower in patients with family history. In addition, multivariate analysis showed that p21 expression was an adverse independent prognostic factor for gastric cancer. These results indicated that low expression of p21 contributed to the good prognosis of gastric cancer patients with family history of cancer. However, the exact mechanism is unclear, and further study is needed.

A limitation of our study is that it has relied on self-reported family history, and the family history information was not confirmed pathologically. However, we confirmed the family history by asking patients' relatives in order to reduce the probability of under-reports or over-reports. Secondly, we did not investigate genetic mutations for MSI or CDH1.

In conclusion, our study showed that the prognosis of gastric cancer patients with a family history of cancer was better than that of patients without a family history. Given the association of p21 expression and family history of cancer, this result may facilitate further development of agents targeting p21 expression and clinical trials evaluating the role of these agent in gastric cancer patients with a family history of cancer.

## MATERIALS AND METHODS

### Patients

From 2000 to 2008, 1805 patients with histologically confirmed primary gastric adenocarcinoma underwent curative gastrectomy at the Department of Gastric Cancer and Soft Tissue Sarcoma Surgery, Fudan University Shanghai Cancer Center. Exclusion criteria for this study were as follows: (1) surgery status unknown; (2) vital status unknown; (3) uncompleted pathological data. Data were retrieved from operative and pathological reports. Follow-up data were obtained by phone, outpatient visits and our clinical database. Written informed consent was obtained from all patients, and this study was approved by the Ethical Committee of Fudan University Shanghai Cancer Center. Staging was done according to the American Joint Committee on Cancer (AJCC) TNM Staging Classification for Carcinoma of the Stomach (Seventh Edition, 2010). Gastrectomy was performed in accordance with the Japanese Classification of Gastric Carcinoma.

### Immunohistochemical staining

The expression of p21, p53, c-myc, EGFR, and Neu/Her-2 in primary lesions was detected by immunohistochemical staining. All primary antibodies and mouse monoclonal antibodies were purchased from Dako (Hamburg, Germany). The detailed sources, concentrations of antibody and positive site were as follows: anti-p21 (clone SX118), 1:50 dilution, nucleus; anti-p53 (clone DO-7), 1:100 dilution, nucleus; anti-c-myc (clone 9E10), 1:100 dilution, cytoplasm; anti-EGFR (clone E30), 1:50 dilution, cytoplasm or membrane; anti-Neu/Her-2 (clone PN2A), 1:100 dilution, membrane. The staining experiments followed the supplier's instruction. Negative controls were subjected to the same procedure except that the first antibody was replaced by PBS.

### Immunohistochemical staining scores

All slides were evaluated by pathologists without knowledge of patients' clinical data. The percentage of immunoreactive cells was graded on a scale of 0 to 4: no staining was scored as 0, 1-10% of cells stained scored as 1, 11-50% as 2, 51-80% as 3, and 81-100% as 4. The staining intensities were graded from 0 to 3: 0 was defined as negative, 1 as weak, 2 as moderated, and 3 as strong, respectively. An IHS score of 9-12 was considered as strong immunoreactivity (+++), 5-8 as moderate (++), 1-4 as weak (+), and 0 as negative (−). On the final analysis, the cases with a score of less than 1 were considered as negative, and ≥ 1 was regarded as positive. These criteria were based on our previously published results [27].

### Family history evaluation

Family history of cancer was reviewed from the patient interview record. A positive family history of cancer was defined as a history of cancer within second-degree relatives. First-degree relatives were defined as parents, siblings, or offspring, and second-degree relatives were defined as aunts, uncles, nieces, nephews, or grandparents.

### Follow-up

Follow-up of all patients was carried out according to our hospital's standard protocol (every three months for at least 2 years, every six months for the next 3 years, and after 5 years every 12 months for life). The check-up items included physical examination, tumor-marker examination, ultrasound, chest radiography, computed tomographic scan, and endoscopic examination. The median follow-up time was 72 months for all patients.

### Statistical analysis

The patients' features and clinicopathological characteristics were analyzed using the *X*^2^ test for categorical variables. Five-year survival rate was calculated by the Kaplan-Meier method, and the differences between survival curves were examined with the log-rank test. Independent prognostic factors were examined by the multivariate survival analysis using the Cox proportional hazards model. The accepted level of significance was *P* <0.05. Statistical analyses and graphics were performed using the SPSS 13.0 statistical package (SPSS, Inc., Chicago, IL).

## References

[1] Torre LA, Bray F, Siegel RL, Ferlay J, Lortet-Tieulent J, Jemal A (2015). Global cancer statistics 2012. CA Cancer J Clin.

[2] Chen MJ, Chiou YY, Wu DC, Wu SL (2000). Lifestyle habits and gastric cancer in a hospital-based case-control study in Taiwan. Am J Gastroenterol.

[3] Uemura N, Okamoto S, Yamamoto S, Matsumura N, Yamaguchi S, Yamakido M, Taniyama K, Sasaki N, Schlemper RJ (2001). Helicobacter pylori infection and the development of gastric cancer. N Engl J Med.

[4] Sung NY, Choi KS, Park EC, Park K, Lee SY, Lee AK, Choi IJ, Jung KW, Won YJ, Shin HR (2007). Smoking, alcohol and gastric cancer risk in Korean men: The National Health Insurance Corporation Study. Br J Cancer.

[5] Peleteiro B, Lopes C, Figueiredo C, Lunet N (2011). Salt intake and gastric cancer risk according to Helicobacter pylori infection, smoking, tumor site and histological type. Br J Cancer.

[6] La Vecchia C, Negri E, Franceschi S, Gentile A (1992). Family history and the risk of stomach and colorectal cancer. Cancer.

[7] Yaghoobi M, Bijarchi R, Narod S (2010). Family history and the risk of gastric cancer. Br J Cancer.

[8] Lee WJ, Hong RL, Lai IR, Chen CN, Lee PH, Huang MT (2003). Clinicopathologic characteristics and prognoses of gastric cancer in patients with a positive familial history of cancer. J Clin Gastroenterol.

[9] Han MA, Oh MG, Choi IJ, Park SR, Ryu KW, Nam BH, Cho SJ, Kim CG, Lee JH, Kim YW (2012). Association of family history with cancer recurrence and survival in patients with gastric cancer. J Clin Oncol.

[10] Kawasaki K, Kanemitsu K, Yasuda T, Kamigaki T, Kuroda D, Kuroda Y (2007). Family history of cancer in Japanese gastric cancer patients. Gastric Cancer.

[11] Chan JA, Meyerhardt JA, Niedzwiecki D, Hollis D, Saltz LB, Mayer RJ, Thomas J, Schaefer P, Whittom R, Hantel A, Goldberg RM, Warren RS, Bertagnolli M, Fuchs CS (2008). Association of family history with cancer recurrence and survival among patients with stage III colon cancer. JAMA.

[12] Thalib L, Wedren S, Granath F, Adami HO, Rydh B, Magnusson C, Hall P (2004). Breast cancer prognosis in relation to family history of breast and ovarian cancer. Br J Cancer.

[13] Yatsuya H, Toyoshima H, Mizoue T, Kondo T, Tamakoshi K, Hori Y, Tokui N, Hoshiyama Y, Kikuchi S, Sakata K, Hayakawa N, Tamakoshi A, Ohno Y, Yoshimura T (2002). Family history and the risk of stomach cancer death in Japan: differences by age and gender. Int J Cancer.

[14] Gao Y, Hu N, Han X, Giffen C, Ding T, Goldstein A, Taylor P (2009). Family history of cancer and risk for esophageal and gastric cancer in Shanxi, China. BMC Cancer.

[15] Caldas C, Carneiro F, Lynch HT, Yokota J, Wiesner GL, Powell SM, Lewis FR, Huntsman DG, Pharoah PD, Jankowski JA, MacLeod P, Vogelsang H, Keller G (1999). Familiar gastric cancer: overview and guidelines for management. J Med Genet.

[16] Lee WJ, Lin JT, Lee WC, Shun CT, Hong RL, Cheng AL, Lee PH, Wei TC, Chen KM (1995). Clinicopathologic characteristics of Helicobacter Pylori seropositive gastric adenocarcinoma. J Clin Gastroenterol.

[17] Palli D, Russo A, Ottini L, Masala G, Saieva C, Amorosi A, Cama A, D'Amico C, Falchetti M, Palmirotta R, Decarli A, Mariani Costantini R, Fraumeni JF (2001). Red meat, family history, and increased risk of gastric cancer with microsatellite instability. Cancer Res.

[18] Pedrazzani C, Corso G, Velho S, Leite M, Pascale V, Bettarini F, Marrelli D, Seruca R, Roviello F (2009). Evidence of tumor microsatellite instability in gastric cancer with familiar aggregation. Fam Cancer.

[19] Brandt A, Bermejo JL, Sundquist J, Hemminki K (2008). Age of onset in familial cancer. Ann Oncol.

[20] Moolgavkar SH, Luebeck EG (1992). Multistage carcinogenesis: Population-based model for colon cancer. J Natl Cancer Inst.

[21] Inoue M, Tajima K, Yamamura Y, Hamajima N, Hirose K, Kodera Y, Kito T, Tominaga S (1998). Family history and subsite of gastric cancer: data from a case-referent study in Japan. Int J Cancer.

[22] Williams KP, Reiter P, Mabiso A, Maurer J, Paskett E (2009). Family history of cancer predicts Papanicolaou screening behavior for African American and white women. Cancer.

[23] Wallner LP, Sarma AV, Lieber MM, St Sauver JL, Jacobson DJ, McGree ME, Gowan ME, Jacobsen SJ (2008). Psychosocial factors associated with an increased frequency of prostate cancer screening in men ages 40 to 79 years: The Olmsted County Study. Cancer Epidemiol Biomarkers Prev.

[24] Humpel N, Magee C, Jones SC (2007). The impact of a cancer diagnosis on the health behaviors of cancer survivors and their family and friends. Support Cancer Cancer.

[25] Patterson F, Wileyto EP, Segal J, Kurz J, Glanz K, Hanlon A (2010). Intention to quit smoking: Role of personal and family member cancer diagnosis. Health Educ Res.

[26] Corso G, Pedrazzani C, Marrelli D, Pascale V, Pinto E, Roviello F (2009). Correlation of microsatellite instability at multiple loci with long-term survival in advanced gastric carcinoma. Arch Surg.

[27] Liu X, Cai H, Huang H, Long Z, Shi Y, Wang Y (2011). The prognostic significance of apoptosis-related biological markers in Chinese gastric cancer patients. PloS One.

